# Peat-Free Composted Bark and Wood Fiber Blends for Container Ornamentals: Species-Dependent Growth and Quality Responses

**DOI:** 10.3390/plants15193062

**Published:** 2026-10-07

**Authors:** Akinson Tumbure, Munno Prasad, Eoghan Corbett, Zoia A. Awan, Dónall Flanagan, Michael T. Gaffney

**Affiliations:** 1 Horticulture Development Department, Teagasc, Ashtown Food Research Center, D15 DY05 Dublin, Ireland; eoghan.corbett@teagasc.ie (E.C.); zoia.awan@teagasc.ie (Z.A.A.); donall.flanagan@teagasc.ie (D.F.); 2 Department of Chemical Sciences, University of Limerick, V94 T9PX Limerick, Ireland; munoo.prasad@ul.ie

**Keywords:** woody ornamentals, sedge, sustainable growing media, nutrients, potted plants

## Abstract

Identification of environmentally sustainable alternative materials or material blends capable of effectively replacing peat in ornamental horticulture is a significant global challenge. The present study involved five separate experiments with three growing media treatments consisting of two composted bark and wood fiber mixes (at 80:20 and 60:40; bark:wood fiber) and pure peat as control. Five different ornamental plants, namely *Leptospermum scoparium* ‘Martinii’, *Carex morrowii* ‘Everglow’, *Cotoneaster frigidus* ‘Cornubia’, *Viburnum tinus* ‘Eve Price’, and *Hypericum × hidcoteense* ‘Hidcote’, were evaluated during the study. Results revealed that all the plant species performed well and produced higher aesthetically ranked plants in the two bark mixtures as compared to control (peat), except *Leptospermum scoparium* ‘Martinii’. The same bark mixes (80:20 and 60:40; bark:wood fiber) produced significantly higher biomass than peat for *Viburnum tinus* ‘Eve Price’ (>53.9%) and *Hypericum × hidcoteense* ‘Hidcote’ (>17.7%), while for *Carex morrowii ‘*Everglow*’* and *Cotoneaster frigidus* ‘Cornubia’, the bark mixes were comparable to peat. However, *Leptospermum scoparium* ‘Martinii’ peat produced significantly higher biomass than the bark mixes. These bark mixes produced more biomass than peat under *Viburnum tinus* ‘Eve Price’ and *Hypericum × hidcoteense* ‘Hidcote’ even though the peat had significantly higher pre-planting mineral nitrogen (>×2.29) and available phosphorus (>×1.54) than the bark mixes. These results show that composted bark and wood fiber growing media can effectively be used as an alternative to peat for the woody ornamentals and sedge species tested, although responses were species-dependent.

## 1. Introduction

The European Union (EU) holds the largest market share of 31% in the cut flowers and ornamental potted plants industry [1]. The total value of the Irish gardening market is estimated to be €966 million (2023), which is segmented into 45% garden products, 23% landscaping services, 19% outdoor and flowering plants, 10% fresh-cut flowers, foliage and wreaths, and 3% indoor potted plants [2]. Soilless culture is often employed to produce high-quality plants. The global horticulture industry was estimated to use about 116 mm^3^ of soilless growing media in 2022 and this figure is projected to rise by at least 250% in the year 2050 [3]. As in other parts of the EU, nurseries have traditionally relied on peat as an optimal growing medium for producing high-quality plants, supported by nearly a century of extensive research and continuous optimization of peat-based cultivation systems [4]. For decades, peat extraction from natural peat bogs has supported the horticulture industry in the EU because of its availability, biological stability, high water holding capacity and its ability to produce high-quality plants when pH-corrected and fertilized. However, a future reduction in or complete cessation of peat use in horticulture is anticipated because of policy changes and environmental concerns arising from peat extraction. These issues include degradation of natural wetlands that has led to biodiversity loss, increased greenhouse gas emissions, poor water quality and reduced recreational space [5]. Currently, the horticultural industry in the EU is transitioning from peat use, albeit gradually. This is because no single alternative material has been identified that can universally replace peat. Consequently, blending locally available multiple materials and adapting agronomic management practices are essential for achieving suitable growing conditions and plant quality [4,6].

Potential materials that can be used as growing media should be sustainable and available while satisfactorily supporting good plant growth compared to peat. Forestry products such as wood fiber and barks are good candidates for use as growing media components because they are renewable resources which can be obtained in a sustainable manner [7]. A recent study by Tumbure et al. [6] quantified forest resources in Ireland and revealed that over 132,000 m^3^ of raw bark and over 356,000 m^3^ of wood chips and sawdust are potentially available annually, providing a practical rationale for evaluating these materials as components of peat-free sustainable growing media. Materials such as tree barks are a low-value forestry by-product which can be turned into a higher value one when used as a growing media component [8]. In Ireland there are few publicly available research reports on the agronomic performance of locally available materials such as barks and wood fiber for growing woody ornamentals and sedges. Although relevant work is being undertaken within the sector, much of the research is not publicly available, as it forms part of private companies’ internal research and development.

Results from previous studies conducted in Ireland assessing peat-alternative growing media found that a 50:40:10 (composted bark:wood fiber:perlite) mix supported strawberry growth and yields similarly to peat [9]. Although these findings demonstrate the potential of such growing-media mixtures, the continuous supply of nutrients through fertigation may mask the effects of the growing medium, particularly in container-grown ornamentals [4]. In container-grown nursery production plant nutrient supply is more dependent on interactions of the growing media with water, fertilizer, and the plant. Here, the growing media interactions may therefore become more important and previous research has concluded that in order to avoid agronomic losses peat-alternative blends should still contain about 25–50% peat in the mix [4]. Some researchers have set an upper limit for wood fiber in growing media at 40% [10]. Other researchers corroborate that the partial replacement of peat is currently the most economically sustainable and eco-efficient pathway for production of pot-based ornamentals [11].

Physico-chemical properties of composted bark vary widely depending on the source/plant species of the bark, type of physical processing, and the composting method used. For instance, an early composting study involving pine and eucalyptus bark reported that composted pine bark was more suitable as a growing media component than composted eucalyptus bark [12]. The same authors attributed the unsuitability of eucalyptus bark to its high EC, associated with high Ca content, which is common in hardwoods. Research from the 1980s had previously noted that composted barks from hardwoods also have a tendency to immobilize micronutrients and result in reduced micronutrient uptake by plants [13]. Other earlier studies showed that 100% uncomposted *Pinus radiata* bark could be used to grow ornamental plants such as ferns, rubber plant, Kalanchoe and Aralia with comparable results to Irish peat [14].

In Ireland most forest resources that are harvested annually are from the softwood Sitka spruce (*Picea sitchensis*) providing most of the country’s bark and wood fiber. Despite this, publicly available information on how the locally obtained composted bark performs in container-based ornamental production is lacking. The objective of this research was therefore to evaluate whether composted bark mixed with wood fiber as peat substitutes supports good growth of woody ornamentals and a sedge in comparison to a peat-based growing media. The null hypothesis was that there would be no significant difference in plants grown in composted bark and wood fiber mixes as peat substitutes and those grown in peat in terms of aboveground biomass, plant height, and spread. To further understand the macronutrient interactions in the peat and no-peat growing media, the available macronutrients (N, P, and K) were assessed at the beginning and end of crop growth.

## 2. Results

### 2.1. Agronomic Performance and Marketability of Nursery Plants

#### 2.1.1. Visual and Aesthetic Scores

**Figure 1:**
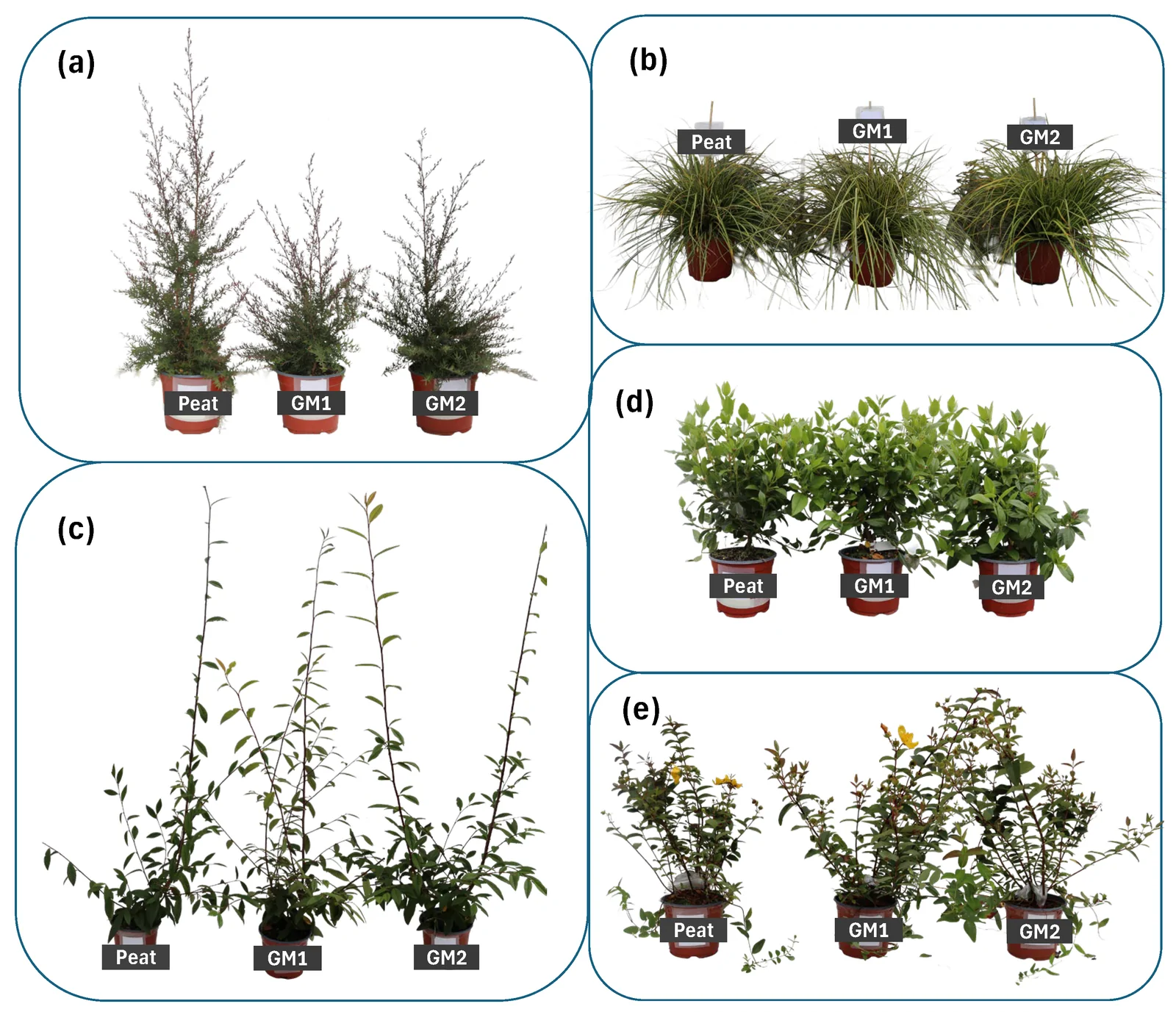
Visual appearance of (**a**) *Leptospermum scoparium* ‘Martinii’, (**b**) *Carex morrowii ‘*Everglow*’*, (**c**) *Cotoneaster frigidus* ‘Cornubia’, (**d**) *Viburnum tinus* ‘Eve Price’, and (**e**) *Hypericum × hidcoteense* ‘Hidcote’ growing on peat (control) and composted bark:wood fiber growing media (80:20 and 60:40 for GM1 and GM2 respectively).

The visual appearance of the five species is presented in Figure 1. At the time of biomass harvest, growth differences were immediately visible across growing media in the *Leptospermum scoparium* ‘Martinii’ species (Figure 1a) where the peat control seemed to have grown bigger sized plants compared to the bark mixes (GM1 and GM2). However, for *Carex morrowii* ‘Everglow’, *Cotoneaster frigidus* ‘Cornubia’, and *Viburnum tinus* ‘Eve Price’ visual differences were not immediately visible before individual scoring was done (Figure 1b–d). At the same time, for *Hypericum × hidcoteense* ‘Hidcote’ the bark mixes seemed to have visually grown bigger sized plants compared to the peat control (Figure 1e). 

**Figure 2:**
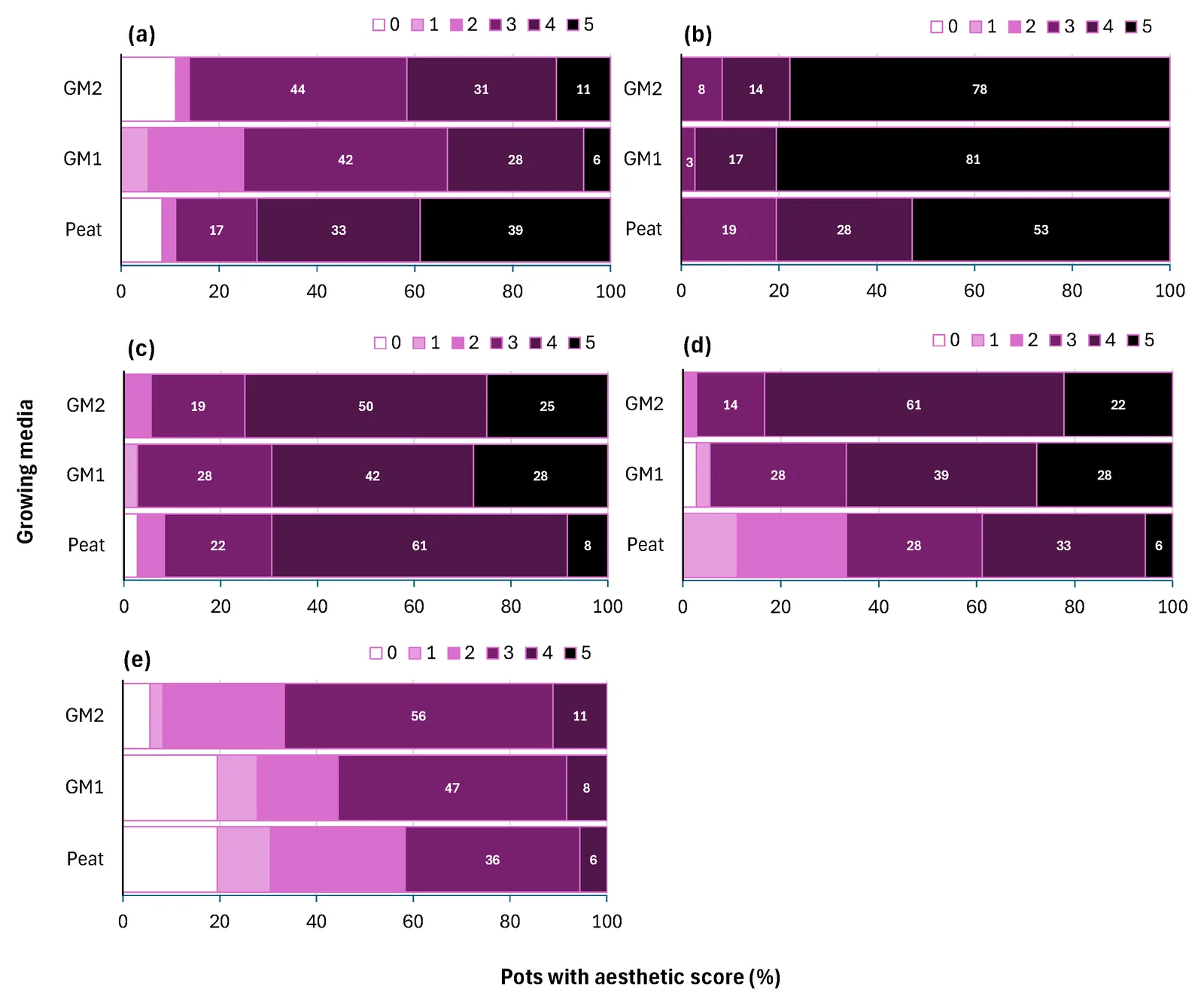
Aesthetic score frequency distribution of (**a**) *Leptospermum scoparium* ‘Martinii’, (**b**) *Carex morrowii* ‘Everglow’, (**c**) *Cotoneaster frigidus* ‘Cornubia’, (**d**) *Viburnum tinus* ‘Eve Price’, and (**e**) *Hypericum × hidcoteense* ‘Hidcote’ growing on peat (control) and composted bark:wood fiber growing media (80:20 and 60:40 for GM1 and GM2 respectively). Numbers inside bars indicate percentages of marketable plants with scores 3 to 5 (*n* = 36).

Generally, the GM1 and GM2 growing media produced high-scoring plants across all the species tested (Figure 2) with at least 94% marketable plants (score ≥ 3) for *Carex morrowii* ‘Everglow’, *Cotoneaster frigidus* ‘Cornubia’, and *Viburnum tinus* ‘Eve Price’. *Carex morrowii* ‘Everglow’ performed well across all the treatments, producing 100% marketable plants in the peat, GM1 and GM2 growing media (Figure 2b). At least 92% of the 100% marketable *Carex morrowii* ‘Everglow’ plants were ranked as either perfect (score 5) or near perfect (score 4) for the bark mixes (GM1, GM2) compared with 81% in peat. Except for *Leptospermum scoparium* ‘Martinii’, all other plant species tested had a greater proportion of higher ranked plants for bark mixes (GM1, GM2) compared with peat. All growing media treatments tested with *Cotoneaster frigidus* ‘Cornubia’ produced at least 92% marketable plants while about 69–75% had high scores (score 4, 5) (Figure 2c). For *Viburnum tinus* ‘Eve Price’, only 67% of plants grown in peat were marketable, while those of the bark mixes maintained higher percentages of at least 92% marketable plants (Figure 2d). The proportion of marketable plants for *Leptospermum scoparium* ‘Martinii’ ranged between 76 and 89% across all the growing media treatments. However, for this species, more plants (72% of total) grown in peat were highly scored (score 4/5) compared to the bark mixes which had a range of 33–42% of plants with score 4/5. *Hypericum × hidcoteense ‘*Hidcote’ was generally the poorest-performing species in terms of aesthetic scores regardless of growing media treatment. Here, the species had total marketable plants ranging from 42 to 67% across all the growing media (Figure 2e) while the lowest scores were observed for peat.

#### 2.1.2. Plant Growth Attributes

Plant survival rates for *Leptospermum scoparium* ‘Martinii’, *Cotoneaster frigidus* ‘Cornubia’, and *Viburnum tinus* ‘Eve Price’ were high across all treatments (89–100%), while *Hypericum × hidcoteense* ‘Hidcote’ had slightly lower survival rates of 81% in peat and GM1 (Table 1). There were no significant differences in plant height of *Leptospermum scoparium* ‘Martinii’ and *Cotoneaster frigidus* ‘Cornubia’ across all the treatments. For *Viburnum tinus* ‘Eve Price’ and *Hypericum × hidcoteense* ‘Hidcote’, GM2 produced plants with significantly higher height than peat. For plant spread, there were no significant differences amongst the growing media for *Cotoneaster frigidus* ‘Cornubia’ and *Hypericum × hidcoteense* ‘Hidcote’. However, for *Viburnum tinus* ‘Eve Price’, the GM1 and GM2 growing media produced plants with significantly greater plant spread than peat. The opposite was true for the *Leptospermum scoparium* ‘Martinii’ species where plants in peat had greater plant spread.

**Figure 3:**
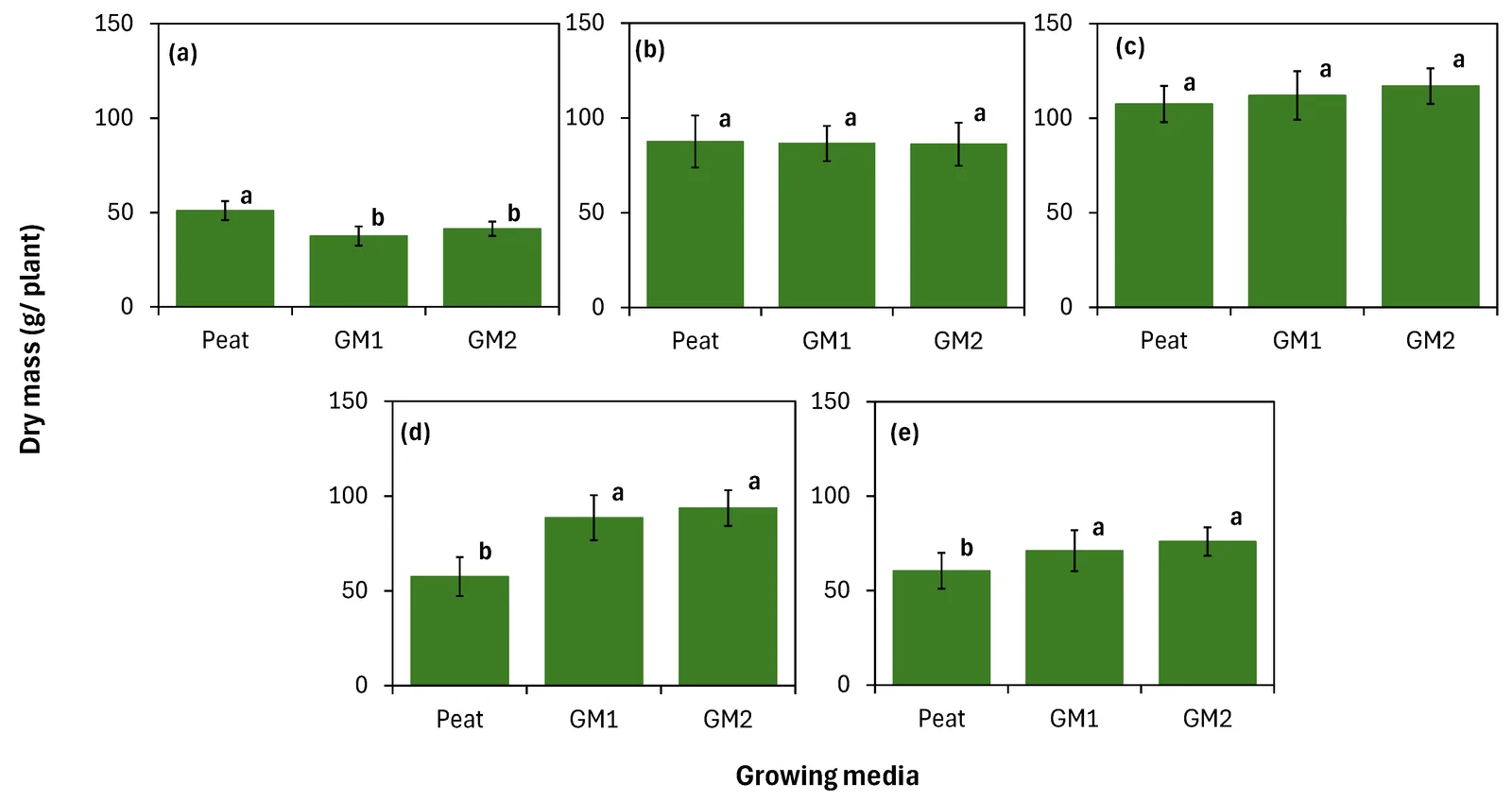
Dry mass production of (**a**) *Leptospermum scoparium* ‘Martinii’, (**b**) *Carex morrowii* ‘Everglow’, (**c**) *Cotoneaster frigidus* ‘Cornubia’, (**d**) *Viburnum tinus* ‘Eve Price’, and (**e**) *Hypericum × hidcoteense* ‘Hidcote’ grown in various growing media. Peat (control) and 80:20 and 60:40 composted bark:wood fiber for GM1 and GM2 respectively. Error bars are standard deviations and different letters above bars represent significant differences at α = 0.05.

For *Carex morrowii* ‘Everglow’ and *Cotoneaster frigidus* ‘Cornubia’, both bark mixes (GM1 and GM2) did not differ significantly from peat in biomass production. The same bark mixes produced significantly higher biomass than peat for *Viburnum tinus* ‘Eve Price’ (>53.9% higher, Figure 3d) and *Hypericum × hidcoteense* ‘Hidcote’ (>17.7% higher, Figure 3e). Among all the species tested, the peat only outperformed the bark mixes in terms of biomass production for *Leptospermum scoparium* ‘Martinii’ only. Here, peat produced significantly more biomass (>18.8%) compared to the composted bark mixes (Figure 3a).

### 2.2. Selected Properties of the Growing Media Pre- and Post-Growth

#### 2.2.1. Electrical Conductivity and pH

In potted plant production, the pH of growing media is an important property to maintain in the optimal range (pH 5.5–6.5) for good plant-nutrient availability [15]. The EC is equally important as it is a measure of soluble salts, which, when high, affect plant nutrient uptake by causing osmotic stress on a growing plant. The composted bark treatments (GM1 and GM2) had pre-planting bulk densities similar to that of peat, with lab compacted bulk density (dry basis) of 119, 160, and 127 g/L for peat, GM1 and GM2 respectively. However, there were significant differences in the initial pH and EC of the growing media. The peat and GM1 were similar in terms of initial pH and EC, while GM2 had significantly higher pH than peat and significantly lower EC than peat (Table 2). After the growth of all species tested, EC did not differ significantly among growing media, while significant differences in pH of the growing media existed. The pH of the growing media containing composted bark remained similar to pre-planting values after growth of *Cotoneaster frigidus* ‘Cornubia’ and *Carex morrowii* ‘Everglow’; following growth of *Viburnum tinus* ‘Eve Price’, *Leptospermum scoparium* ‘Martinii’ and *Hypericum × hidcoteense* ‘Hidcote’ there was a general increase in pH (range 0.4–0.9). The post-growth trend in pH was comparable across all the plant species where peat significantly had a lower pH value than the bark mixes (GM1, GM2) except for *Hypericum × hidcoteense* ‘Hidcote’ where pH of GM1 was comparable to peat.

#### 2.2.2. Available Macronutrients (N, P, and K)

**Figure 4:**
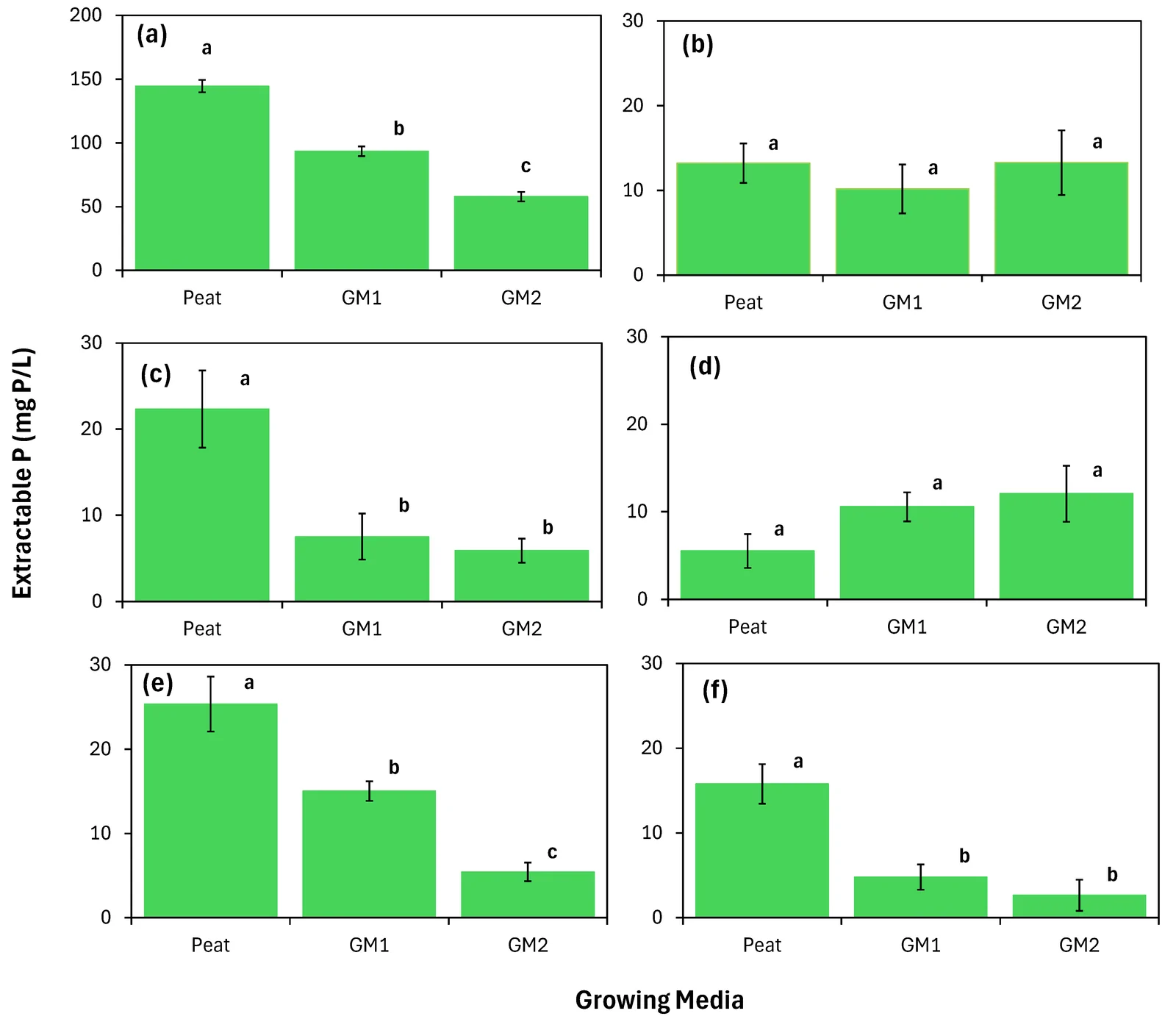
Available phosphorus in various growing media at (**a**) pre-planting, and post-harvesting stages for (**b**) *Leptospermum scoparium* ‘Martinii’, (**c**) *Cotoneaster frigidus* ‘Cornubia’, (**d**) *Viburnum tinus* ‘Eve Price’, (**e**) *Hypericum × hidcoteense* ‘Hidcote’, and (**f**) *Carex morrowii* ‘Everglow’. Error bars are standard deviations (*n* = 6). Different letters above bars represent significant differences at α = 0.05.

The standard practice of adding essential macronutrients N, P, and K as slow-release fertilizers in container-grown ornamentals is to ensure that plant-available nutrient levels remain at optimum levels for plant growth throughout the long growing season. However, different types of growing media chemically and biologically modify the rhizosphere matrices, which in turn affects plant nutrient availability. In the current study, the availability of selected macronutrients N, P, and K was an indication of these matrix-modifying effects. Extractable P in the peat growing medium (peat) was initially high at 144 mg P/L compared to the GM1 and GM2 growing media (at least 54% more) (Figure 4a). The pre-planting extractable P was 93 and 58 mg P/L for GM1 and GM2 respectively, which was near the optimum range (50–80 mg P/L). The initial trend amongst the growing media was peat > GM1 > GM2, which was maintained after growth of *Cotoneaster frigidus* ‘Cornubia’, *Hypericum × hidcoteense* ‘Hidcote’ and *Carex morrowii* ‘Everglow’, while there were no significant differences amongst the growing media after growth of *Leptospermum scoparium* ‘Martinii’ and *Viburnum tinus* ‘Eve Price’ (Figure 4b,d). The range of extractable P post-growth of all the species was extremely low (3–25 mg P/L).

**Figure 5:**
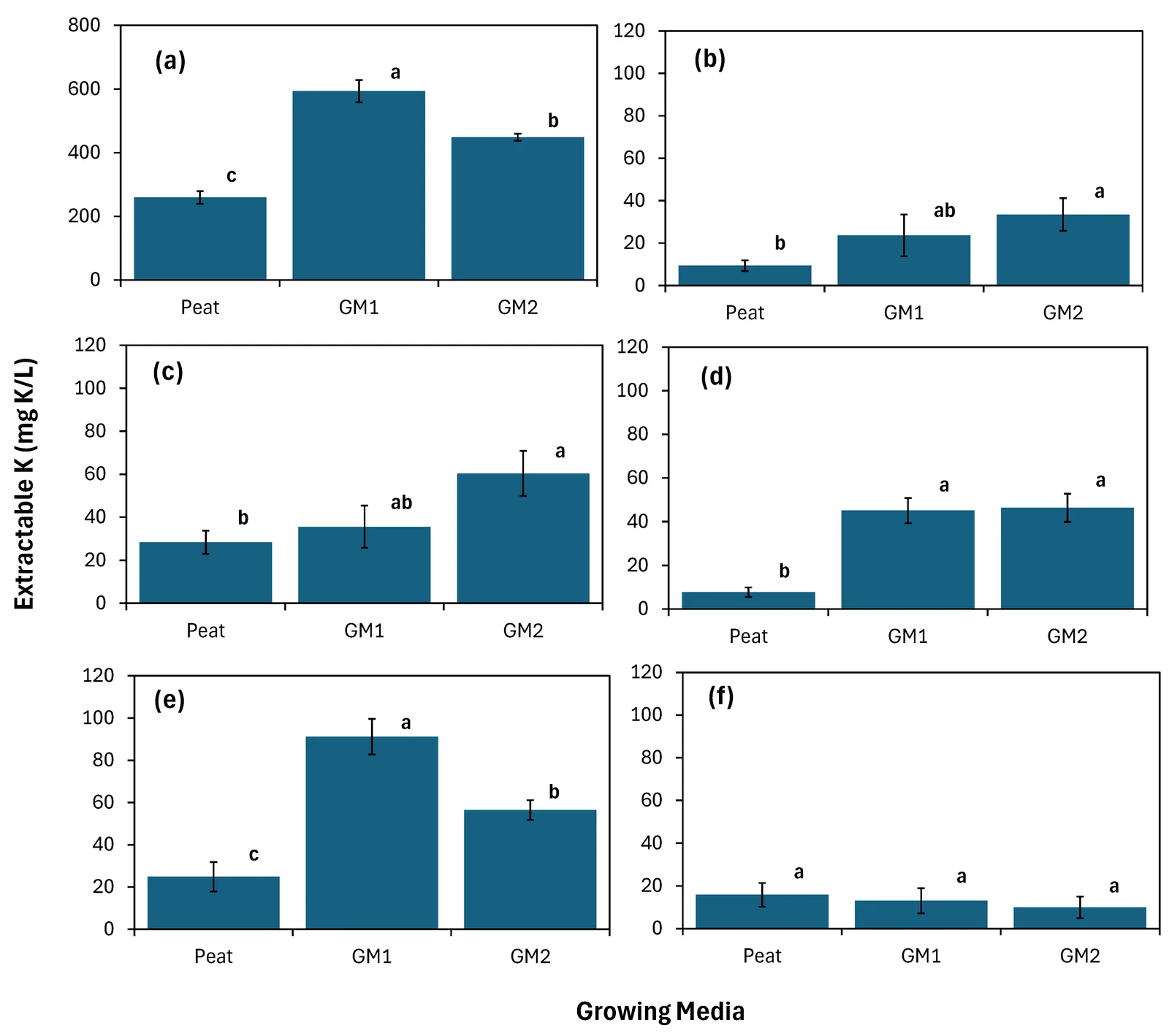
Available potassium in various growing media at (**a**) pre-planting and post-harvesting stages for (**b**) *Leptospermum scoparium* ‘Martinii’, (**c**) *Cotoneaster frigidus* ‘Cornubia’, (**d**) *Viburnum tinus* ‘Eve Price’, (**e**) *Hypericum × hidcoteense* ‘Hidcote’, and (**f**) *Carex morrowii* ‘Everglow’. Error bars are standard deviations (*n* = 6). Different letters above bars represent significant differences at α = 0.05.

The pre-planting extractable K in the growing media followed the trend GM1 > GM2 > peat with the bark mixes having at least 72% higher extractable K than peat (Figure 5a). The peat and GM2 media had pre-planting extractable K values that were near the optimum 200–400 mg K/L. However, across all species, after plant growth, the extractable K decreased to less than 100 mg K/L. Generally, the peat had the lowest extractable K after growth of *Leptospermum scoparium* ‘Martinii’, *Cotoneaster frigidus* ‘Cornubia’, *Viburnum tinus* ‘Eve Price’, and *Hypericum × hidcoteense* ‘Hidcote’, while after growth of *Carex morrowii* ‘Everglow’ there were no significant differences in extractable K amongst the growing media treatments.

**Figure 6:**
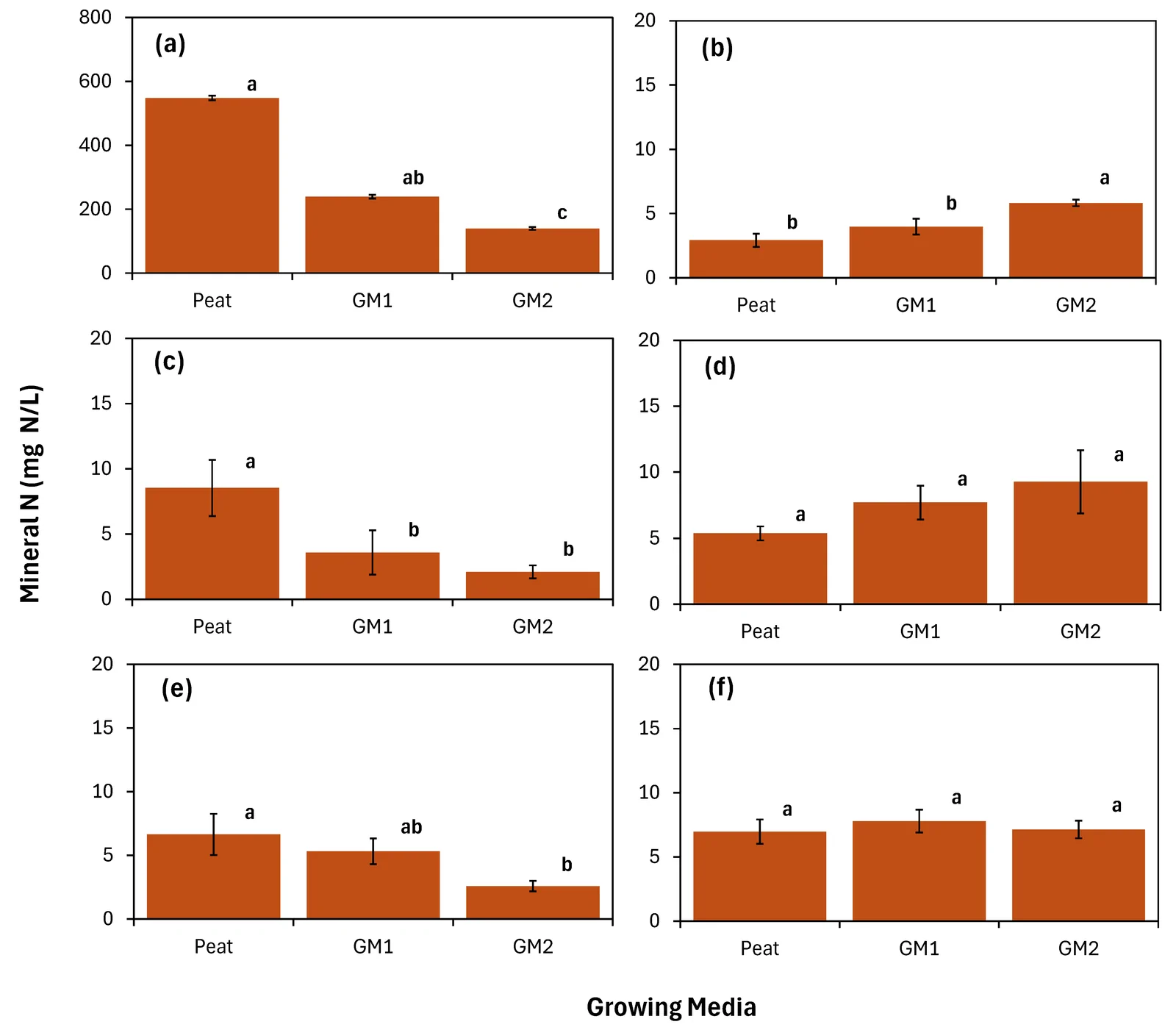
Mineral nitrogen in various growing media at (**a**) pre-planting and post-harvesting stages for (**b**) *Leptospermum scoparium* ‘Martinii’, (**c**) *Cotoneaster frigidus* ‘Cornubia’, (**d**) *Viburnum tinus* ‘Eve Price’, (**e**) *Hypericum × hidcoteense* ‘Hidcote’, and (**f**) *Carex morrowii* ‘Everglow’. Error bars are standard deviations (*n* = 6). Different letters above bars represent significant differences at α = 0.05.

An almost similar trend to available P was observed for mineral N in the pre-planting growing media where the peat growing medium had the highest mineral N content which was at least x2.29 that of the other formulated media (Figure 6a). The pre-planting mineral N content of peat was exceptionally high at 548 mg N_min_/L while the composted bark mixes were closer to the optimum range (100–200 mg N_min_/L). After the growth of all species, the N_min_ levels had reduced to very low levels (<10 mg N_min_/L). After *Viburnum tinus* ‘Eve Price’ and *Carex morrowii* ‘Everglow’ growth there were no significant differences in mineral N across all the treatments (Figure 6d,f).

## 3. Discussion

### 3.1. Agronomic Performance and Marketability of Nursery Plants

The material composition of growing media affects how supplied nutrients interact with media chemical surfaces, microbes and plant roots and as a result how nutrients remain available in synchrony to plant development. When nutrient supply is out of synchrony with plant development, nutrient losses can occur; for example, high available N may be leached out during heavy rain before the plant can take it up. On the opposite side, applied N may become unavailable when it is quickly taken up by microbes which rapidly multiply due to presence of easily degradable C bio-compounds from growing media. Nutrients such as P may be ‘locked’ up in plant-unavailable forms especially as growing media pH increases (pH > 7) and there is some Fe content. The overall quality of potted ornamentals therefore relies heavily on how the material composition of the growing media affects nutrient, water and air exchange. *Carex morrowii* ‘Everglow’ and *Cotoneaster frigidus* ‘Cornubia’ performed well across all the growing media, producing 100% and ≥92% marketable plants, respectively, in all the growing media tested. The fact that biomass production was similar across all the growing media demonstrates the adaptability of both these species to various growing media conditions. *Carex morrowii* ‘Everglow’ is moderately tolerant to salinity stress as observed by Akpinar [16] and tolerant to low moisture (<10% volumetric water content) in potted plant settings [17]. The initially high available N content of peat may therefore have been less detrimental to *Carex morrowii* ‘Everglow’. This characteristic has made *Carex* spp. good candidates for N removal to counteract eutrophication in high-N-contaminated peatlands [18]. Similarly, the results of our study on *Cotoneaster frigidus* ‘Cornubia’ are consistent with previous work in which *Cotoneaster* spp. grew similarly well in 100% composted bark and 100% municipal solid waste compost despite having substantially different initial ECs of 400 and 4800 µS/cm respectively [19].

Of all the species tested, peat produced significantly greater biomass than the composted bark mixes for biomass production only in *Leptospermum scoparium* ‘Martinii’; peat had a greater aesthetical score and significantly greater plant spread. In terms of nutrient requirements, *Leptospermum scoparium* ‘Martinii’ is described as a species that has low nutrient needs and is known to grow well even in waterlogged soils [20,21]. Nevertheless, *Leptospermum scoparium* ‘Martinii’ responds vigorously to high N nutrient supply [22]. Because peat medium had significantly higher initial N_min_ than the composted bark mixes, this is likely to have been the main contributor to the observed differences.

For *Viburnum tinus* ‘Eve Price’ and *Hypericum × hidcoteense* ‘Hidcote’, GM2 produced plants with significantly higher plant height and aboveground biomass than peat. It is interesting to note that previous studies by Guérin et al. [23] using the same species and peat-alternative growing media reported that the tallest *Viburnum tinus* ‘Eve Price’ plants corresponded with high water content in the growing media while EC and cation exchange capacity (CEC) had no relationship to plant height. At the same time, *Viburnum tinus* ‘Eve Price’ is noted to be tolerant of drought conditions and variation in growing media chemical properties [24,25]. It is probable that the GM2 growing media performed the best in terms of plant height, spread, and biomass because of increased plant water availability associated with its higher wood fiber content likely to degrade during plant growth compared to the GM1 media. Recently, Durand and Michel [26] reported that commercially supplied wood fibers showed an increase in water retention attributable to the formation of new fine pores during degradation in a 6-week aerobic biodegradation experiment that mimicked natural potted conditions. Potted media containing wood fiber may have root zone temperatures differences compared to peat. Nevertheless, *Viburnum tinus* ‘Eve Price’ may also be sensitive to the high available N observed in peat. A study by Martín et al. [27] using an N^15^ tracer showed that *Viburnum tinus* could not regulate ammonium absorption and therefore higher concentrations of ammonium may be toxic to the plant.

*Hypericum* spp. roots are also known to also exhibit antimicrobial properties which may reduce nitrifying bacteria populations and therefore nitrification of ammonium [28]. Such an effect could result in reduced nitrate leaching and ensure higher supply of N within a pot during the growing season.

For *Hypericum × hidcoteense* ‘Hidcote’, the reduced plant heights and biomass in peat may be attributed to the initially high mineral N (585 mg N/L). High available N, especially in the form of ammonium, may hamper *Hypericum* spp. root and overall growth [29,30,31]. These factors could have been significant in affecting aesthetic scores and agronomic growth of both *Viburnum tinus* ‘Eve Price’ and *Hypericum × hidcoteense* ‘Hidcote’ where the control peat underperformed relative to the composted bark mixes (GM2, GM1). It is also likely that the composted bark blends had N mineralization in good synchrony to plant growth which gave rise to better aboveground biomass in composted bark blends compared to peat for *Viburnum tinus* ‘Eve Price’ and *Hypericum × hidcoteense* ‘Hidcote’.

### 3.2. Selected Properties of the Growing Media Pre- and Post-Growth

The recommended generic fertilization of peat when growing long-term shrubs (over 1 year) is up to 6 g/L of a controlled-release fertilizer [32]. If Osmocote 5™ 12–14M (16:3.5:10, N:P:K) is used as a base macrofertilizer at 5 g/L, then this corresponds to an estimated 800, 175, and 500 mg of total N, P, and K respectively per liter of media. In this study, about 860 mg N/L was added to peat, and roughly 900 mg N/L was added to the bark mixes (GM1 and GM2). The slight increase in N applied to composted bark mixes (+5% more) compared to peat was to ensure adequate N supply in case of N immobilization because of the high C:N ratio in wood fiber. The immobilization of N in wood fiber and composted barks is well known [4,6]. The assumption that the composted bark and wood fiber mixes could have reduced available N is consistent with the composted bark mixes being initially within or close to the optimum range (120–200 mg N_min_/L [33]) despite the slightly higher total N application rate of bark mixes (5% more N) while that of the peat medium was exceptionally high at 548 mg N_min_/L. The surface chemistry of the composted bark + wood fiber mixes may therefore have reduced the proportion of applied N that was immediately available relative to peat. While uncomposted bark is reported to have high N immobilization potential [34], when composted, this potential is reduced. However, the N immobilization potential of wood fiber remains high if the wood fiber is not pretreated/stabilized in some way.

The total K applied in bark mixes was 30% less than that in peat because the bark raw material contained significant amounts of available K (340 mg K/L). However, despite this reduction in K application which was meant to balance out available K among treatments, at the pre-planting stage, the bark mixes had significantly more (72%) exchangeable K than peat. While pre-planting exchangeable K was optimum for peat, for the composted bark mixes (GM1 and GM2), it was higher than the 120–300 mg K/L optimum [33]. However, the high K in the composted bark mixes did not appear to be associated with obvious negative effects in the species tested.

Pre-planting available P for the composted bark mixes was within the expected optimum range (40–90 mg P/L; [33]), while for peat it was high (144 mg P/L). Despite the fact that pre-planting values for K were higher than optimum for composted bark mixes and that pre-planting N and P values were higher than optimum for peat, all the growing media tested had EC values that were within the 300–500 μS/cm optimum range [33].

Post-plant growth concentrations of available N, P and K were very low in all the growing media treatments and for all the five species that were tested (3–25 mg P/L, <100 mg K/L, and <10 mg N_min_/L). This suggests that most of the initially supplied macronutrients had either been used up by the plant or leached through the media. The measured available macronutrient levels at the end of the experiments were now below the reported ranges for optimal plant growth. However, plant nutrient deficiency symptoms were not evident at time of harvest. The reduction in available N, P, and K in media post-plant growth coincided with at least 32% reduction in the EC compared to pre-planting values for peat and GM1 growing media for all species. For GM2 growing media and post-*Leptospermum scoparium* ‘Martinii’, *Cotoneaster frigidus* ‘Cornubia’, and *Viburnum tinus* ‘Eve Price’, there was a +6 to +19% increase in EC compared to pre-planting values, suggesting that the less-available macronutrients could have been replaced by other ions mineralized during the growth of these species. The slight increase in pH, post-growth of the tested species, may also be consistent with changes in the balance of soluble cations. A probable alkaline cation is Ca^2+^ that is usually in high proportions in bark [35] and is known to be slowly released in bark [36]. However, available Ca was not measured in this case since the standard (CAT) extraction procedure uses CaCl_2_.

### 3.3. Research Limitations and Future Research

The current research focused on selected macronutrients and micronutrients were not studied. Though total micronutrients were applied at sufficient levels, growing media matrix effects may affect availability of some micronutrients and further studies to assess the availability of micronutrients in composted bark and wood fiber growing media are required.

Having the commercial peat medium pre-fertilized meant that the peat control had a slightly different complex fertilizer blend than the bark mixes. The major difference was however for the nitrogen as all N applied to peat was through slow-release fertilizers, while for bark mixes calcium ammonium nitrate and urea were used which are more readily soluble. This difference did not contribute to more mineral N in bark mixes compared to peat as observed in pre-planting analysis. Nevertheless, future studies on the effects of fertilizer type are recommended especially in composted bark-based growing media where reduced mineral N was observed despite adding more soluble N before plant growth.

Available N, P, and K were measured only at the pre-planting and biomass-harvesting stages while observed differences in plant quality and biomass are a result of how available nutrients changed during the whole plant-growth period. Future studies should focus on continuous N, P, and K measurements using ion-selective electrodes/solid-state meters or employing destructive sampling to assess nutrient availability in alternative media at more time points during plant growth.

The limited number of species employed in this study was through selection of some of the more popular ornamentals grown in Ireland. There is need to validate results obtained in this study with additional ornamental crops and under commercial nursery conditions. Future studies should investigate the suitability or goodness of fit of alternative growing media in current production facilities. This is an often overlooked but important aspect which encompasses whether new alternative blends have different handling requirements which require adjustments to irrigation setups and automated production/potting systems.

## 4. Materials and Methods

### 4.1. Growing Media Components and Experimental Design

All materials that were used for making the growing media treatments were locally sourced in Ireland. Except for the commercial peat growing media (peat) that was purchased already fertilized, the other raw materials were fertilized before being used as growing media components.

The rates of Osmocote 5™ 12–14M (from ICL group Ltd., Tel Aviv, Israel), calcium nitrate, urea, and dolomitic lime applied to the wood fiber and composted bark are shown in Table 3. The Osmocote 5™ 12–14M has a 16:3.5:10 (N:P:K) macronutrient ratio, calcium nitrate contained 15.5% N, and urea contained 46% N. Extra micronutrients were not added because Osmocote 5™ 12–14M has sufficient micronutrient content when applied at 4 g/L (0.3% Fe, 0.012% Zn, 0.01% B, 0.05% Mn, 0.015% Cu and 0.01% Mo). Agricultural lime/dolomitic lime was added to mainly correct the acidic pH of the composted bark component. The wood fiber component was not limed to avoid alkaline conditions during plant growth because other researchers have noted that growing media with around 50% or more wood fiber may rapidly increase pH within 5 weeks of plant growth [15].

The peat was purchased pre-fertilized with 0.5 g/L PG Mix (12:14:24, N:P:K) from Yara Ireland, Loughbeg, Ireland, 0.25 g/L PG micromix from Yara Ireland, Loughbeg, Ireland, 0.5 g/L Osmocote 5™ 5–6M (16:3.5:10 (N:P:K)), 4.5 g/L Osmocote 5™ 12–14M, and 4 g/L lime. The peat medium was used as a control treatment and 2 treatments of growing media were mixed as (*v*/*v*) 80% composted bark + 20% wood fiber (GM1), and 60% composted bark + 40% wood fiber (GM2). The estimated applied macronutrients were 0.86–0.91 g N/L, 0.14–0.21 g P/L, and 0.41–0.6 g K/L for the growing media mixes (Table 4).

The three treatments were replicated six (6) times and arranged as a randomized complete block design. Each treatment within a block consisted of six 3 L pots arranged in a line and each treatment line was randomized in the block. This design was employed to set up a total of 5 side-by-side experiments, each with *Leptospermum scoparium* ‘Martinii’, *Carex morrowii* ‘Everglow’, *Cotoneaster frigidus* ‘Cornubia’, *Viburnum tinus* ‘Eve Price’, or *Hypericum × hidcoteense* ‘Hidcote’ as the plant test species and a total of 108 pots/experiment. The lab compacted bulk densities (g/L) of the growing media on a dry basis were 119 ± 1.5 (peat), 160 ± 1.9 (GM1), and 127 ± 2.7 (GM2).

### 4.2. Experiment Establishment and Management

Three-liter pots were manually filled with the same quantity of growing media taking care to maintain a uniform compression of the different types of media in each pot. Young plants of *Leptospermum scoparium ‘*Martinii*’*, *Cotoneaster frigidus* ‘Cornubia’, *Viburnum tinus* ‘Eve Price’, *Hypericum × hidcoteense* ‘Hidcote’, and *Carex morrowii* ‘Everglow’ were obtained from local nurseries. The respective plugs were planted into the growing media treatments at one plant per pot on 7 August 2024. The pots were placed on an open-air pad with a surrounding 2 m tall windbreak. Irrigation was automated through a programmable irrigation controller (Hunter Industries, San Marcos, Calif, USA), delivering water through J-hook drippers (2 L/h) at timed intervals typically of 5 min duration, between 1 and 3 times a day. The number of irrigation events was manually adjusted according to seasonal conditions.

### 4.3. Sampling and Data Collection

Aesthetic scoring of individual plants was performed by one assessor after 11 months in July 2025, where leaf condition/color, density of plant, and growth rate were assessed and scored on a scale of 0–5. The ranking system was based on 0 (dead plant), 1 (4 identified faults/1 severe fault), 2 (3 identified minor faults/1 severe fault), 3 (2 identified minor faults), 4 (1 identified minor fault), and 5 (no identified faults). Examples of identified faults included stunted growth, diseased leaves or leaves showing nutrient deficiencies, and a less dense plant with fewer leaves/branches. If a single fault was severe then the score would be relegated to a 1 or 2, which is a range classified as unmarketable. Scores 3–5 represented plants that were good enough to be retailed. Although the scoring is subjective to the researcher as it is qualitative, it is still a robust way to rank plants as would be done in a natural commercial setting. Previous studies have employed such an aesthetic ranking system in one way or another [37,38,39].

Measurements of plant height and height were recorded for each plant pot. A tape measure was used to measure plant height from the pot level to the highest part of the plant. Pot level was used as the starting point to avoid media-level differences due to growing media compaction/shrinkage differences that could have occurred during plant growth. Plant spread (width) was measured as the horizontal distance from the tips of the two furthest opposite branches. For this measurement, branches that were not naturally standing or growing downwards were not used. Plant height and spread of *Carex morrowii* ‘Everglow’ was not measured because it is a less important factor as the cultivar was bred for compactness (US Patent No. PP30466 [40]).

All potted plants were individually harvested for aboveground biomass. Each potted plant was cut at the point where the stems joined the growing media and bagged into perforated plastics. The bagged plants were weighed for fresh mass using a tarred scale to subtract the mass of the perforated plastic container. Each bagged plant was then dried to a constant mass in an oven at 75 °C before being reweighed to record each individual dry mass.

After harvesting the plants, the growing media from each treatment line (6 pots) and in each block were mixed and prepared as a composite sample; this was then subsampled to give a total of 6 sample replicates for each treatment. These samples were analyzed for lab compacted bulk density, pH, EC and CaCl_2_/DTPA-extractable N, P, and K. For pH and EC, this was determined after shaking growing media in DI water (1:5 *v*/*v*) for one hour [41,42]. To analyze extractable macronutrients, extraction was carried out using CaCl_2_/DTPA solution [43] and analysis of extractable P, K, nitrate/nitrites and ammonium was performed using a Lachat autoanalyzer (8500 Series 2) from Hach Lange GmbH, Düsseldorf, Germany.

### 4.4. Statistical Analysis

All data were analyzed using the IBM SPSS Statistics package (version 29.0.2.0(20)) for each experiment. Data on plant biomass, plant height and width were analyzed separately for each test species using a Mixed Effects Linear model (MELM) with the within-block pseudo-replicates assigned as random effects and growing media types as fixed effects. The first-order autoregressive covariance structure was employed to account for microclimate differences along a line of pseudo-replicates of the same treatment in a block. Where significant differences were observed between growing media treatments (α = 0.05), pairwise mean comparison was done using the Bonferroni confidence interval adjustment. Assumptions of linearity and homoscedasticity were analyzed by plotting residuals vs. predicted graphs; normality of residuals was analyzed using QQ plots and independence analyzed using the Durbin–Watson test.

Data on pH, EC, and extractable N, P, and K in growing media was checked for normality using the Shapiro–Wilk test and homogeneity of variance was analyzed using the Levene test. Normally distributed data was analyzed using a one-way analysis of variance and where there were significant differences, separation of means was done using Tukey’s HSD. For non-normal data non-parametric tests were done using the independent samples Kruskal–Wallis test.

## 5. Conclusions

Composted bark mixed with wood fiber supported biomass production of woody ornamentals (*Cotoneaster frigidus* ‘Cornubia’, *Viburnum tinus* ‘Eve Price’, *Hypericum × hidcoteense* ‘Hidcote’) and a sedge (*Carex morrowii* ‘Everglow’) similarly to or greater than those observed in peat-based growing medium. However, in the *Leptospermum scoparium* ‘Martinii’ experiment, bark and wood fiber mixes had significantly reduced biomass compared to peat. These results show that Sitka spruce composted bark and wood fiber mixes can have comparable performance in terms of biomass and aesthetic plant quality of *Cotoneaster frigidus* ‘Cornubia’ and *Carex morrowii* ‘Everglow’ and sometimes superior performance when *Viburnum tinus* ‘Eve Price’ and *Hypericum × hidcoteense* ‘Hidcote’ are considered. While composted bark mixes might have good potential as a peat replacement, responses were species-dependent, and peat supported greater vigor in *Leptospermum scoparium* ‘Martinii’ despite a broadly similar proportion of marketable plants. Given that potential availability of composted bark in Ireland is about 132,000 m^3^, high-bark-containing growing media mixes may be reserved for selected ornamentals that are proven to result in comparable-to-peat plant vigor for the professional sector. Further studies employing more varied ornamental species, continuous N, P, and K measurements, and peat-free multi-material growing media mixes are recommended to attain the goal of peat replacement in Irish professional horticulture.

## Figures and Tables

**Table 1:** Plant survival rate and growth attributes of selected species planted in various growing media.

Growing Media	Plant Survival Rate (%) n = 36	Plant Height (cm)	Plant Spread (cm)
*L. scoparium* ‘Martinii’			
peat	91.7	55.3 ^a^ ± 9.0	43.5 ^a^ ± 6.4
GM1	100	50.5 ^a^ ± 10.5	36.6 ^b^ ± 5.6
GM2	88.9	50.6 ^a^ ± 9.5	39.2 ^b^ ± 4.8
Sign ^1^		ns	***
*C. frigidus* ‘Cornubia’			
peat	97.2	134.2 ^a^ ± 17.1	96.9 ^a^ ± 18.1
GM1	100	127.8 ^a^ ± 22.6	99.3 ^a^ ± 19.6
GM2	100	130.9 ^a^ ± 20.0	98.4 ^a^ ± 19.2
Sign		ns	ns
*V. tinus* ‘Eve Price’			
peat	100	35.3 ^b^ ± 6.2	37.1 ^b^ ± 8.1
GM1	97.2	37.7 ^ab^ ± 6.7	46.9 ^a^ ± 6.2
GM2	100	39.1 ^a^ ± 6.5	48.4 ^a^ ± 5.6
Sign		*	***
*H. hidcoteense* ‘Hidcote’			
peat	80.6	42.0 ^b^ ± 9.8	69.8 ^a^ ± 12.9
GM1	80.6	47.5 ^a^ ± 8.2	69.4 ^a^ ± 10.3
GM2	94.4	47.4 ^a^ ± 9.1	74.5 ^a^ ± 8.5
Sign		**	ns

^1^ Sign—significance, ns—not significant (*p* > 0.05), * *p* ≤ 0.05, ** *p* ≤ 0.01, *** *p* ≤ 0.001, figures with different superscript letters are significantly different from each other along a column of the same species. Numbers after ± are standard deviations.

**Table 2:** The pH and EC (1:5 media:DI water) of the growing media at the pre-planting and post-harvesting stages.

	pH					
Media	Pre-planting	Post-*L. scoparium*	Post-*C. frigidus*	Post-*V. tinus*	Post-*H. hidcoteense*	Post-*C. morrowii*
peat	4.5 ^b^ ± 0.1	5.5 ^b^ ± 0.3	4.7 ^b^ ± 0.8	5.5 ^b^ ± 0.1	6.0 ^b^ ± 0.8	5.6 ^b^ ± 0.4
GM1	6.3 ^ab^ ± 0.04	7.2 ^a^ ± 0.1	6.2 ^a^ ± 0.9	7.0 ^a^ ± 0.04	7.2 ^ab^ ± 0.1	6.3 ^a^ ± 0.1
GM2	6.5 ^a^ ± 0.1	7.0 ^a^ ± 0.2	6.5 ^a^ ± 0.1	6.9 ^a^ ± 0.1	7.2 ^a^ ± 0.04	6.4 ^a^ ± 0.04
Sign ^1^	*	*	*	*	*	*
	EC (μS/cm)					
Media	Pre-planting	Post-*L. scoparium*	Post-*C. frigidus*	Post-*V. tinus*	Post-*H. hidcoteense*	Post-*C. morrowii*
peat	392.3 ^a^ ± 50.8	198.1 ^a^ ± 33.9	252.3 ^a^ ± 43.8	213.8 ^a^ ± 28.3	173.5 ^a^ ± 45.9	207.7 ^a^ ± 43.8
GM1	365.3 ^a^ ± 6.0	203.8 ^a^ ± 31.7	226.8 ^a^ ± 27.8	246.0 ^a^ ± 31.0	196.2 ^a^ ± 50.9	188.0 ^a^ ± 27.6
GM2	224.3 ^b^ ± 17.1	238.7 ^a^ ± 80.6	246.6 ^a^ ± 49.2	268.3 ^a^ ± 50.7	208.2 ^a^ ± 76.7	173.2 ^a^ ± 16.0
Sign	*	ns	ns	ns	ns	ns

^1^ Sign—significance, ns—not significant (*p* > 0.05), * *p* ≤ 0.05. Numbers after ± are standard deviations, *n* = 3 for pre-planting, *n* = 6 for post-harvest values. Figures with different superscript letters are significantly different from each other along a column.

**Table 3:** Fertilizer rates used for raw components.

Material	Osmocote 5 ™ 12–14M	Calcium Nitrate	Urea	Dolomitic Lime
Wood fiber	4.5 g/L	0.4 g/L	0.2 g/L	-
Composted bark	4 g/L	1.2 g/L	0.2 g/L	6 g/L

**Table 4:** Calculated macronutrient loading rates in final mixes.

Media	N (g/L)	P (g/L)	K (g/L)	Mg (g/L)	Ca (g/L)
Peat	0.86	0.21	0.60	0.53	0.86
GM1	0.91	0.14	0.41	0.61	1.23
GM2	0.90	0.15	0.42	0.47	0.94

## Data Availability

Data is unavailable upon request to authors.

## Abbreviations

The following abbreviations are used in this manuscript:

EU	European Union
EC	Electrical Conductivity
N_min_	Mineral Nitrogen
CEC	Cation Exchange Capacity
DTPA	Diethylene Triamine Pentaacetic Acid
